# Assessment of Cyberostracism and Personality Inventory In First Year University Students In Turkey

**DOI:** 10.1093/eurpub/ckac130.062

**Published:** 2022-10-25

**Authors:** FN Öznur Muz, A Ünsal, D Arslantaş, A Kılınç, M Tözün

**Affiliations:** 1 Public Health, Eskisehir Osmangazi University Faculty of Medicine, Eskisehir, Turkey; 2 Public Health, İzmir Katip Çelebi University Faculty of Medicine, İzmir, Turkey

## Abstract

**Background:**

Being ignored, one of the most common problems among young people, can occur not only in face-to-face communication but also through social media communication tools over the internet and is called cyberostracism (CO). The effects of cyberostracism on people are at least as effective as in real social life and can cause a wide variety of affective disorders. It is thought that the family, environmental factors and the individual's own personality traits effectively control such negative emotions. This study aims to evaluate the CO level and personality types of students who have just started university in Turkey.

**Methods:**

This cross-sectional study was conducted in 2021 spring semester and 3148 students in the first year of their university in Turkey constituted the study group. To evaluate the CO levels of the students, CO Scale (min-max score 14-70) and to evaluate the personality type, Ten Item-Personality Inventory (TIPI) was used. The questionnaire prepared in accordance with literature was filled out online by the students. Mann Whitney U, Kruskal Wallis analyses and Multiple Linear Regression was used.

**Results:**

In the study, 1847 (62.5%) were female and the mean age was 19.9±1.8 years. The mean score obtained from the CO scale was 21.1±8.1; 41.8% of the participants had the Agreeableness personality type. Male gender, extended family, not good at face-to-face communication with friends, creating a membership by hiding their identity in social media and being ignored in social media were predictive for CO (F: 69.176, R2: 0.172, p < 0.001) was shown in multiple linear regression.

**Conclusions:**

Distance education programs during the pandemic period have limited the face-to-face communication of young people, causing them to spend more time in cyberspace. Personality type has been an important factor affecting the level of cyberostracism by determining our behaviour when exposed to difficult life events.

**Key messages:**

- Young people exposed to cyberostracism, may enter dangerous environments and groups that they think will be easily accepted in their real lives.
- It is thought that young people should be guided to use social media more consciously in the future in order to prevent cyberostracism.

